# Supplementary material for: Revisiting density-dependent fecundity in schistosomes using sibship reconstruction
Source: PLoS Negl Trop Dis. 2021 May 13;15(5):e0009396. doi: 10.1371/journal.pntd.0009396 (PMC8148369; doi:10.1371/journal.pntd.0009396)
Supplement: S1 Text — Text A. Model variants with interactive terms permitting the severity of density dependence to vary with the other measured covariates. Fig A. Coefficient of density dependence, β1, estimated from data on Schistosoma mansoni egg counts and numbers of female worms inferred by sibship reconstruction. In panels 1 and 2 each point indicates, respectively, the point estimate and upper 95% confidence limit of the coefficient of density dependence estimated by repeatedly fitting a log-linear regression model to 1,000 datasets. Each dataset was generated by sampling from the posterior distribution of the inferred number of female worms, N, assuming a different value Nmax for the upper bound of the uniform prior distribution of N. The box and whiskers depict the median, interquartile range and the 2.5th and 97.5th percentiles of the estimates. Values less than 1 indicate statistical support for density-dependent fecundity. Table A. Coefficient estimates from the log-linear statistical model fitted to the Schistosoma haematobium data. Coefficient estimates are arithmetic means obtained by repeatedly re-fitting the model to 1,000 datasets. Each dataset was generated by sampling from the posterior distribution of the inferred number of female worms, N, assuming a different value Nmax for the upper bound of the uniform prior distribution of N. The coefficients β0, β1, γ3, and γ4 were statistically significant, showing that worm fecundity was higher in Pemba Island and in children, and showing evidence for density-dependent fecundity. The reference levels used for each factor variable were Pemba Island (γ2), child (γ3), 2012 (γ4) and male (γ5). The averaged ±95%CIs across all Nmax values were β0 (2.96, 3.94), β1 (3.04, 4.22), γ2 year 2016 (2.51, 4.17), γ3 Unguja (1.37, 2.97), γ4 adult (1.87,3.64) and γ5 female (2.39, 3.99). Table B. Coefficient estimates from the log-linear statistical model fitted to the Schistosoma mansoni data. Coefficient estimates are arithmetic means obtain [file pntd.0009396.s001.docx]

# Supporting Information

Revisiting density-dependent fecundity in schistosomes using sibship reconstruction

M. Inês Neves^1,2*^, Charlotte M Gower^2^, Joanne P Webster^1,2^ and Martin Walker^1,2,^

^1^ Department of Pathobiology and Population Sciences, Royal Veterinary College, University of London

^2^ London Centre for Neglected Tropical Disease Research, Imperial College London Faculty of Medicine, London

* corresponding author [mneves@rvc.ac.uk](mailto:mneves@rvc.ac.uk)

**Text A. Model variants with interactive terms permitting the severity of density dependence to vary with the other measured covariates.**

In addition to the additive log-linear regression models (see Eqn. 1 in the main text), we fitted models with interactive terms permitting the severity of density dependence to vary with the other measured covariates. The general form of this model variant is given by

| $\log\left( \mathbf{Y}+1 \right)=\beta_{0}\mathbf{+}\beta_{1}\log\left( \mathbf{N} \right)+\boldsymbol{\gamma X+\delta Z+}\boldsymbol{e}$, | (**S1**) |
| --- | --- |

where $\beta_{0}$, $\beta_{1}$, $\mathbf{N}$, $\boldsymbol{\gamma}$, $\mathbf{X}$ and $\boldsymbol{e}$ are defined in the main text, $\mathbf{Z}$ is a matrix of interaction terms between $\log\left( \mathbf{N} \right)$ and $\mathbf{X}$ and $\boldsymbol{\delta}$ is a corresponding vector of coefficients. The components of $\boldsymbol{\delta}$ permit additive adjustments to the severity of density dependence for different covariate levels. We fitted this alternative model structure to both datasets (*Schistosoma haematobium* in Zanzibar and *S. mansoni* in mainland Tanzania) using the regression calibration approach described in the main text, also repeating the sensitivity analysis using different values of $N_{\max}$. We compared the frequency with which this model was preferred over the simpler model using a likelihood ratio test, for *S. haematobium* in Zanzibar (Table C1) and *S. mansoni* in mainland Tanzania (Table C2).

**
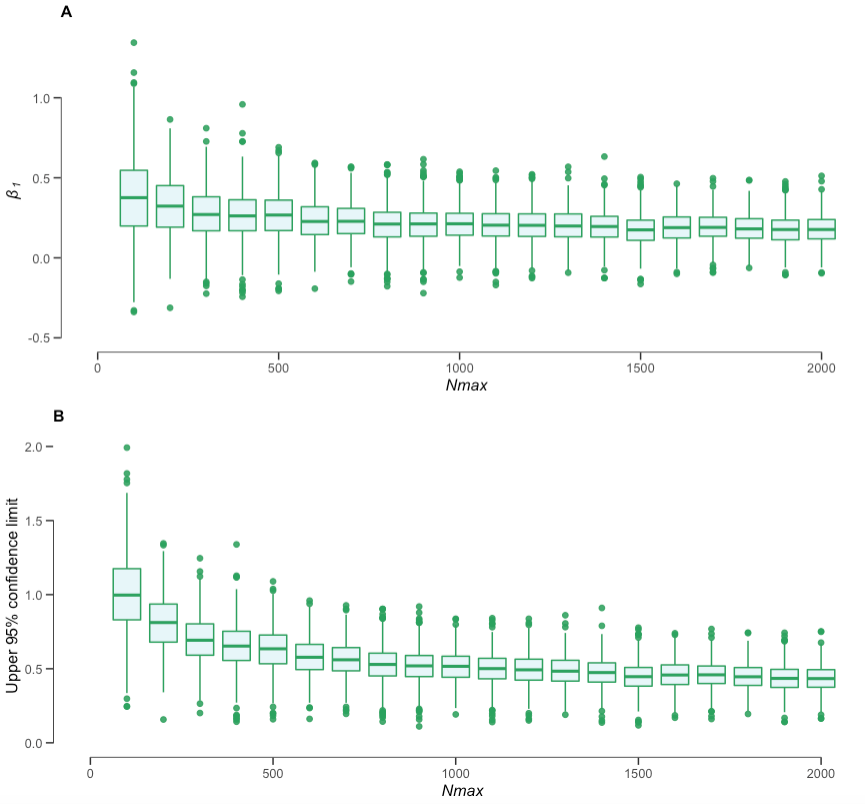
**

**1**

**
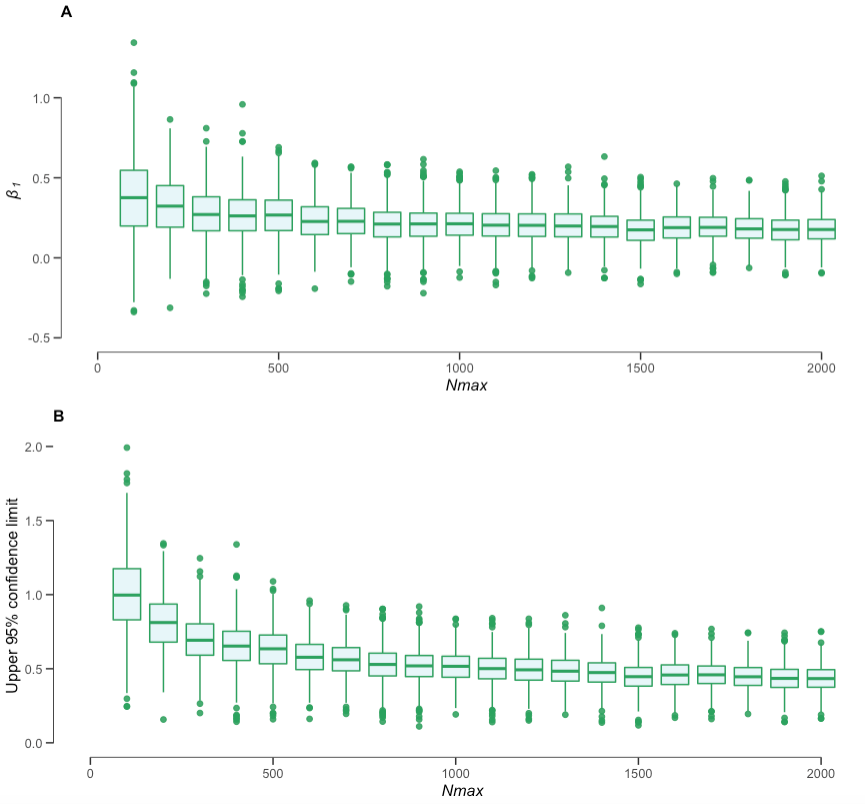
**

**2**

**Fig A**. **Coefficient of density dependence,** $\boldsymbol{\beta}_{\boldsymbol{1}}$**, estimated from data on *Schistosoma mansoni* egg counts and numbers of female worms inferred by sibship reconstruction.** In panels **1** and **2** each point indicates, respectively, the point estimate and upper 95% confidence limit of the coefficient of density dependence estimated by repeatedly fitting a log-linear regression model to 1,000 datasets. Each dataset was generated by sampling from the posterior distribution of the inferred number of female worms, $N$, assuming a different value $N_{\max}$ for the upper bound of the uniform prior distribution of $N$. The box and whiskers depict the median, interquartile range and the 2.5^th^ and 97.5^th^ percentiles of the estimates. Values less than 1 indicate statistical support for density-dependent fecundity.

**Table A. Coefficient estimates from the log-linear statistical model fitted to the *Schistosoma haematobium* data.** Coefficient estimates are arithmetic means obtained by repeatedly re-fitting the model to 1,000 datasets. Each dataset was generated by sampling from the posterior distribution of the inferred number of female worms, $N$, assuming a different value $N_{\max}$ for the upper bound of the uniform prior distribution of $N$. The coefficients $\beta_{0}$, $\beta_{1}$, $\gamma_{3}$, and $\gamma_{4}$ were statistically significant, showing that worm fecundity was higher in Pemba Island and in children, and showing evidence for density-dependent fecundity. The reference levels used for each factor variable were Pemba Island ($\gamma_{2}$), child ($\gamma_{3}$ ), 2012 ($\gamma_{4})$ and male ($\gamma_{5}).$ The averaged ±95%CIs across all $N_{\max}$ values were $\beta_{0}$ (2.96, 3.94), $\beta_{1}$ (3.04, 4.22), $\gamma_{2}$year 2016 (2.51, 4.17), $\gamma_{3}$ Unguja (1.37, 2.97), $\gamma_{4}$adult (1.87,3.64) and $\gamma_{5}$female (2.39, 3.99).­­­


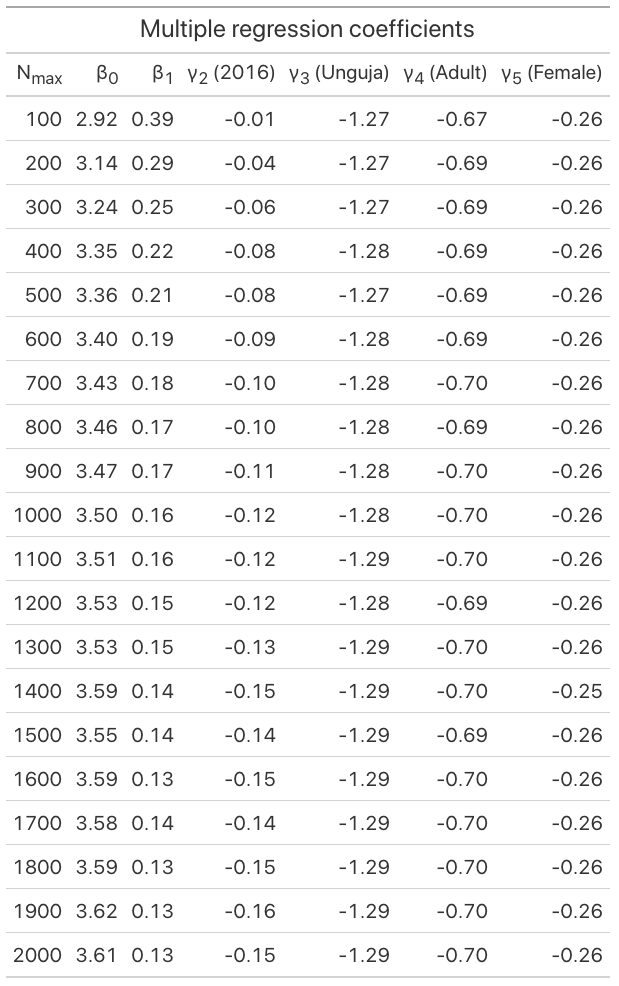


**Table B. Coefficient estimates from the log-linear statistical model fitted to the *Schistosoma mansoni* data.** Coefficient estimates are arithmetic means obtained by repeatedly re-fitting the model to 1,000 datasets. Each dataset was generated by sampling from the posterior distribution of the inferred number of female worms, $N$, assuming a different value $N_{\max}$ for the upper bound of the uniform prior distribution of $N$. The coefficients $\gamma_{2}$ and $\gamma_{3}$were statistically significant, showing that worm fecundity was higher in 2006 and 2010, and Kisorya school. The reference levels used for each factor variable were 2005 ($\gamma_{2}$), Bukindo ($\gamma_{3}$ ) and male ($\gamma_{4}).$ The averaged ±95%CIs across all $N_{max}$ values were $\beta_{0}$ (0,01,3.62), $\beta_{1}$ (-0.10,4.18), $\gamma_{2}$year 2016 (0.41, 5.65), $\gamma_{2}$year 2010 (1.58, 7.33), $\gamma_{3}$ Kisorya (0.95, 5.97), $\gamma_{4}$female (-0.99, 4.0).


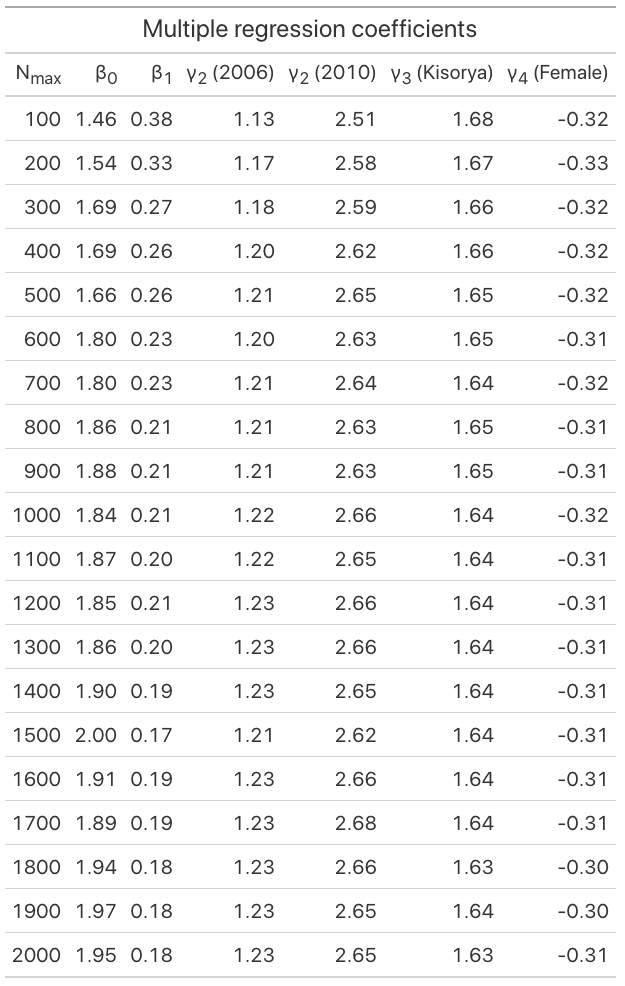


**Table C. Results of likelihood ratio tests comparing the fits of models with additive or interactive terms.** Likelihood ratio tests (LRTs) were used to compare the fits of models including either additive or interactive terms that were re-fitted to 1,000 datasets. Each dataset was generated by sampling from the posterior distribution of the inferred number of female worms, $N$, assuming a different value $N_{\max}$ for the upper bound of the uniform prior distribution of $N$. The frequency with which fitted models including interactive terms were preferred over the simpler model including only additive terms is given in the column labelled “Percentage preference for interactive model”. Results are shown for *Schistosoma haematobium* in Zanzibar in panel **1** and for *S. mansoni* in mainland Tanzania in panel **2**.

**1**


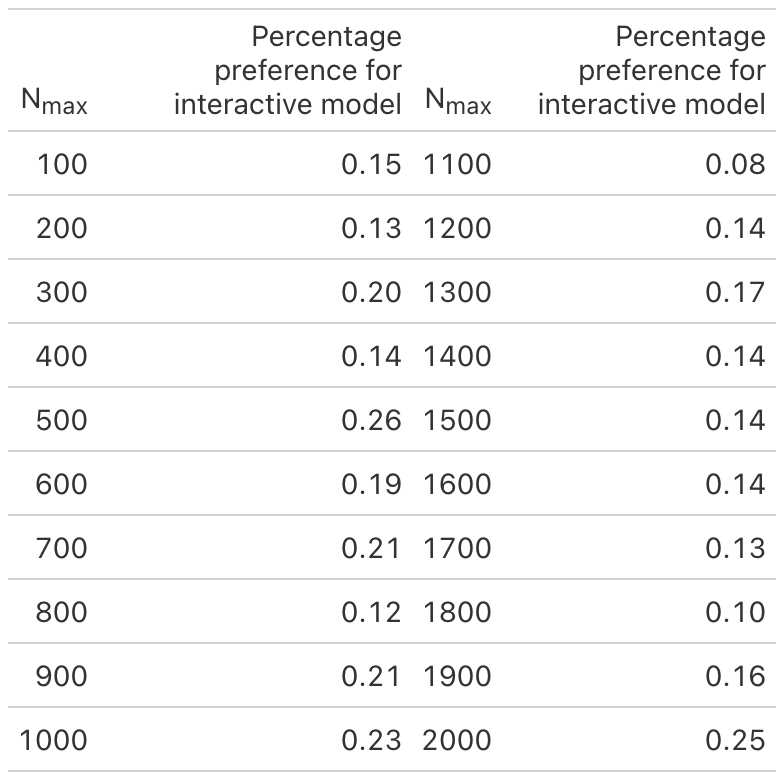


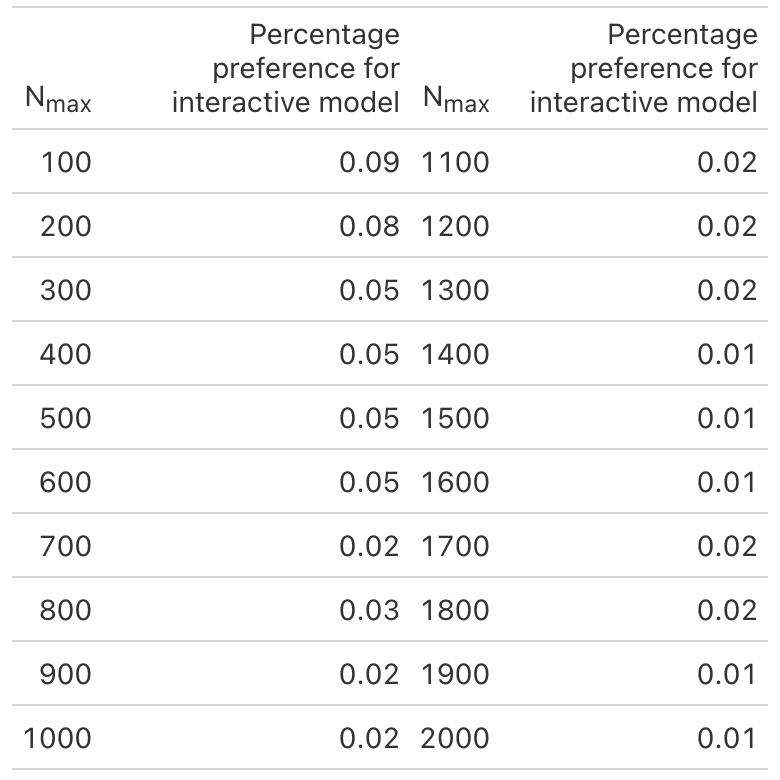


**2**
