# Supplementary material for: Revisiting density-dependent fecundity in schistosomes using sibship reconstruction
Source: PLoS Negl Trop Dis. 2021 May 13;15(5):e0009396. doi: 10.1371/journal.pntd.0009396 (PMC8148369; doi:10.1371/journal.pntd.0009396)
Supplement: Attachment — Submitted filename: response_to_reviewers.docx [file pntd.0009396.s003.docx]

Maria Inês Neves

Department of Pathobiology &

Population Sciences
Royal Veterinary College

Hawkshead Campus

Hawkshead Lane
North Mymms
Hatfield, Herts, AL9 7TA mneves@rvc.ac.uk

Dr. Shaden Kamhawi, Professor Paul Brindley and Professor Peter J. Hotez

Editors-in-Chief

PLoS Neglected Tropical Diseases

March, 2021

Dear Dr. Kamhawi, Professor Brindley and Professor Hotez,

My co-authors and I would like to thank you for considering our original research article entitled ‘Revising density-dependent fecundity in schistosomes using sibship reconstruction’ for publication in *PLoS Neglected Tropical Diseases*. We are extremely grateful to the three Reviewers whose points we have addressed on the subsequent pages of this letter. We are confident that our manuscript has been significantly improved by addressing the Reviewers’ comments.

We very much look forward to hearing from you,

Yours sincerely,

M. Inês Neves (corresponding author on behalf of all co-authors)

Reviewer #1

**Methods**

**Reviewer’s comment 1.1:** The study design addresses the stated objectives. Two existing datasets, ZEST (Zanzibar) and SCORE (Tanzania) were used for S. haematobium and S. mansoni, respectively. Sample sizes are sufficient: ZEST = 224 children and adults before treatment and 214 children and adults after 10 rounds of MDA. SCORE = 151 school children before and during MDA treatment.

My biggest concern is whether the analysis included repeated measures of the same individual and how the authors accounted for that, especially samples collected after treatment (MDA)?

**Authors’ response 1.1:** We thank the Reviewer for this important comment that required clarification in our text. It is possible that the datasets used in the analyses include some, albeit minimal, repeated measures.

In the Zanzibarian (SCORE/ZEST) study, data were recorded as repeated cross sections. That is, at each sample collection time, data from each individual were anonymised and individuals were assigned a new identifier, but this was not matched (for individuals who may have been, by chance, sampled repeatedly) between different years. We acknowledge as a limitation in the revised manuscript that some individuals in the Zanzibarian data may have been sampled repeatedly and that the potential correlation among these repeated measures could not be accounted for.

In the Tanzanian (SCI) study, data were recorded as part of a longitudinal design. However, the percentage of individuals contributing repeated measures was low. Firstly, because there was a high dropout rate during the study, the percentage of individuals followed up in 2006 was 2.5% in one school, and 19% in the other school. In 2010, the corresponding percentages are not known, but they are likely to be lower, since only the children who were 7 or 8 years old in 2005 or 2006 could possibly have been sampled in 2010.

The relevant sections of the revised manuscript now read:

*“An additional limitation related to the nature of data is that we could not account for the possibility that some individuals may have been sampled repeatedly, leading to potential correlation among these repeated measures. For the Zanzibarian (SCORE/ZEST) study, data were recorded as independent cross-sections at each sampling time. That is, at each sample collection time, data from each individual were assigned a new identifier, but this was not matched (for individuals who may have been sampled repeatedly) between different years. Nevertheless, the percentage of individuals contributing repeated measures is likely to be low, because both the participants and the subset of participants contributing samples for population genetic analysis were randomly selected at each year. For the Tanzanian (SCI) study,* *the percentage of individuals sampled in* 2005 *and followed up in 2006 was 2.5% in one school, and 19% in the other school [1]. In 2010, this percentage is likely to be even lower, since only the children who were 7 or 8 years old in 2005 or 2006 could have been resampled in 2010. ”*

**Reviewer’s comment 1.2:** The miracidia used to determine sibship reconstruction had low sample sizes. However, I assume that it is the nature of the sampling schemes for both schistosoma species. In other words, normal numbers expected with egg hatching detection methods from urine and stool samples.

The authors did not include details on the egg hatching technique. Are there differences between egg hatching from urine samples or stool samples? What was the egg hatching success rate? 
Other than my concern mentioned above, the statistical approaches seemed applicable and limitations and assumptions were clearly stated. There are no IRB approved references – however the use of human subjects in this study do not necessarily require and IRB approval. Although, institutional differences might exist.

**Authors’ response 1.2:** We thank the Reviewer for this comment. Egg hatching and filtering from urine samples and stool samples were performed using the same method for both schistosome species – and we have added additional details, and cross-referenced to an earlier paper which describes these in more detail, within our revised text. We would also be happy to add these to the Supplementary Information section if the editors require. Unfortunately, however, the egg hatching rate was not recorded in this study, as the deviation from slide-positive to successful hatching was minimal (in contrast to that which can be found amongst human infection is, for instance, hybridizes or mixed foci regions, which were not the case in either of our regions here). The manuscript now reads as:

*“Egg hatching and isolation of S. mansoni and S. haematobium was performed by concentrating eggs from all infected urine samples by filtration using a Pitchford Funnel, rinsing and transferring into a clean Petri dish containing mineral water and exposing eggs to light to facilitate hatching of miracidia [2].”*

**Results**

**Reviewer’s comment 1.3**: The simpler additive models included indicator variables that was not reported on in great detail (I do understand that it was not the main focus of the paper). However, I suggested a supplementary text box extrapolating on all the significant indicator variables that were presented in Tables S1 and S2. Age differences, location differences, etc. How the indicator variables are influenced by the observed eggs (epg or eggs per 10ml urine)? Etc. 

**Authors’ response 1.3:** We thank the Reviewer for these comments. Indeed, the main focus of the paper were the model coefficients relevant to density-dependent fecundity, but we understand that the other coefficients also provide important information on the epidemiology of schistosomiasis. We have reported *all* coefficient estimates from both models (fitted *to* *S. mansoni* or *S.* *haematobium* data) in Supplementary Table S1 (*S. haematobium*) and Table S2 (*S. mansoni*) as well as indicating which of these coefficients reached statistical significance. However, we have refrained from providing further interpretation of these estimates because these are discussed in greater detail in Gower *et al.* 2017 and in a forthcoming publication from Pennance *et al.* 2021 (*in preparation*).

**Reviewer’s comment 1.4:** The authors also noted that the models inclu­ding interaction terms were less parsimonious. So why did the authors decide to use the simpler additive models? Perhaps they could provide a clear explanation supporting their decision.

**Authors’ response 1.4:** We chose the additive models over the models that included interaction terms because the added complexity of the latter did not produce a sufficiently improved fit to the data to warrant being preferred over the simpler models. This is based on well-established statistical (and philosophical) principles of parsimony. We have clarified the selection of the additive models in the revised manuscript as follows:

“These models did not provide a sufficiently improved fit to the data to warrant being preferred over the simpler more parsimonious additive models, as indicated by likelihood-ratio tests (see Supplementary Information Text S1 and Table S3).”

**Reviewer’s comment 1.5:** The figures and tables seem to be of sufficient quality.

**Authors’ response 1.5:** Thank you for this remark.

**Conclusions**

**Reviewer’s comment 1.6**: The authors conclusions aligned with the data presented and limitations of the models were clearly stated.

**Authors’ response 1.6:** We thank the Reviewer for these appreciative comments.

**Editorial and Data Presentation Modifications**

**Reviewer’s comment 1.7:** Major issues: How did the authors accounted for repeated measures of individual samples used (before and after treatment)?

**Authors’ response 1.7:** We thank the Reviewer for this important comment. We have provided a response to this question in **Authors’ response 1.1.**

**Reviewer’s comment 1.8:** Minor issues:
Minor comments/edits – see specific lines under headings.

Line 52: Perhaps the authors could be more descriptive to the “they” they’re referring to. Should it be “We”?

Line 227: Rephrase (e.g., We found supportive evidence that clearly demonstrated density-dependent fecundity in S. haematobium, the causative agent responsible for urogenital schistosomiasis).
Line 232: Rephrase (e.g., “… and can be extended to other human helminthiases…”
Line 244: Rephrase (e.g., “…A recent study by Gower et al. [37], utilizing the same Tanzaniam S. mansoni dataset, found …”).

Line 255-262: I would suggest re-organizing content in the paragraph(s). Perhaps starting as follow: Contradictory results exist for density-dependent fecundity in S. haematobium. One study analyzed 197 infected cases at autopsy found no significant evidence of density dependence [27]. Whereas a relative recent study… dependence [43].
Line 261: Move up to previous paragraph (line 260).
Line 262: Start sentence with “Firstly, in…”
Line 264: Start sentence with “Secondly, it is possible…”
Line 265: Delete “On the other hand” Perhaps phrase as follow: Although our non-invasive method of exploring density dependence is more indirect (but practicable), it does invoke modeling assumptions (to which… limitation.

Line 313: S. mansoni should be in italics
Line 320: Delete “we believe that”
Line 321: Replace evaluating with evaluate
Line 359: Add “to” (e.g., …be used to explore…)
Line 366: Delete “that”
Line 394: Suggestion: “…schistosomiasis. In addition, we illustrate how our approach…”

Table S1 and Tables S2: I would suggest adding the reference levels for the indicator variables to your table caption. 
Table S3: Caption reads strangely – Perhaps: “… (LRT) at different values of Nmax for A) S. haematobium in Zanzibar and B) S. mansoni in mainland Tanzania.”

**Authors’ response 1.8:** We have amended all the typographical errors identified by the Reviewer and we have reviewed the suggestions for stylistic changes in the Discussion, clarifying the text as required.

**Reviewer’s comment 1.9:** Suggesting a Supplementary text box extrapolating on all the significant indicator variables that were presented in Tables S1 and S2. Age differences, location differences, etc. How the indicator variables are influenced by the observed eggs (epg or eggs per 10ml urine)?

**Authors’ response 1.9:** We have provided a response for this question on **Authors’ response 1.3.**

**Reviewer’s comment 1.10:** Line 145: I wonder if it is possible to briefly explain the specific details on the molecular analysis and sibship reconstruction?

**Authors’ response 1.10:** We thank the Reviewer for this comment, which has also been mentioned by Reviewer #2. We agree with the Reviewer’s point and have added a brief description of the sibship reconstruction method in the Methods section. This paragraph reads:

*“In both studies, the number of unique female S. haematobium and S. mansoni genotypes within each individual, n, was estimated by analysing multiplexed microsatellite genotypic data of individual miracidia (hatched from eggs) using sibship reconstruction methods [36]. Sibship reconstruction is a category of parentage analysis which can be used to estimate the number of parents when genetic data are available on offspring only [34-36, 43]. Essentially, data on neutral genetic markers are used to divide offspring into groups of full siblings (monogamous mating) or groups of full siblings and half siblings (polygamous mating) to reconstruct and identify unique (male and/or female) parental genotypes. Hence the technique has potential tocan be used to as a method of estimateing worm burdens ([44], and see for examples [32, 38, 45].) after statistical adjustment for the number of offspring (here, miracidia) sampled [39].”*

**Reviewer’s comment 1.11:** Line 146 Statistical modeling: General comment for this section: How did the authors account for repeated measures of the same individual (data collection from before and after treatment as explained under lines 121-126)? Individual ID must be included as a random effect if repeated measures of the same individual exist.

**Authors’ response 1.11:** We have responded to this question in **Authors’ response 1.1.**

**Reviewer’s comment 1.12:** Line 180-188: I would suggest this whole section move to methods rather than results. Although, rephrasing and word choice could also improve the fit as results. 

**Authors’ response 1.12:** We agree with the reviewer and have decided to remove this paragraph from the Results, since it had already been described in the Methods.

**Reviewer’s comment 1.13:** Line 186: Start the sentence “We used a…” with Briefly, we used a… Assuming here that the authors explaining the “recently developed statistical approach”.

**Authors’ response 1.13:** We thank the reviewer for this comment. We have decided to keep this sentence as it is, since we have provided the reference which includes the details of the statistical approach.

**Reviewer’s comment 1.14:** Line 217-219: Are there any interesting results that could be reported here from the supplementary outputs? Also, see my minor issues suggestion.

**Authors’ response 1.14:** We thank the Reviewer for this comment. We have provided a response for this question in **Authors’ response 1.3.**

**Reviewer’s comment 1.15:** Line 221-222: If the interaction term models were less parsimonious why did the authors decide to use the simpler additive models?

**Authors’ response 1.15:** We have provided a response to this question in **Authors’ response 1.4.**

**Reviewer’s comment 1.16:** Line 282: What do the authors mean by “obtaining as many accessible parasite stages”? 

**Authors’ response 1.16:** In this case, by “accessible parasite stages”, we are referring to obtaining as many schistosome offspring as possible, i.e. the eggs in faeces or urine which can be hatched and subsequently genotyped. We have clarified this in the revised manuscript as follows:

*“It also reiterates the imperative of sampling effort to obtain as many accessible offspring parasite stages (in this case, miracidia) as possible [39].”*

**Reviewer’s comment 1.17:** Line 306: Perhaps the authors could reiterate the “specific assumptions” here.

**Author’s response 1.17:** We thank the Reviewer for this comment. The specific assumptions and limitations of sibship reconstruction that we are referring to are briefly discussed at the beginning of the same paragraph, and in more detail in our previous work. We have clarified the revised manuscript as follows:

*“In addition to the statistical limitations of the approach used here, there exist a variety of population biological and genetic assumptions not considered here explicitly that may affect the accuracy of sibship reconstruction. These include assumptions of monogamous versus polygamous mating and of Mendelian inheritance and Hardy Weinberg equilibrium of parental genotypes (see [39] for a more detailed discussion). Moreover, the number and type of microsatellite markers used in the analysis and the possible existence of clonal worms within hosts (due to the asexual reproduction of the parasite in snails; but note that cercariae mix in water before infecting hosts which likely mitigates this effect) are also limitations of the sibship reconstruction method. Notwithstanding, the statistical relationship between the estimated number of fecund female worms and the number of unique parental genotypes identified from a finite sample of (miracidial) offspring will be unaffected by the specific assumptions used for sibship reconstruction.”*

**Reviewer’s comment 1.18:** Line 355: In what context does the authors use “the same egg counts measured”? Do the authors infer that the same egg count method is used or is it the variation having different individuals doing the egg counts?

**Author’s response 1.18:** We agree with the Reviewer that this statement was not very clear and it has been removed from the revised manuscript.

**Reviewer’s comment 1.19:** Figure 3: Out of curiosity, what was the criteria for the Nmax limit of 250.

**Authors’ response 1.19:** We thank the Reviewer for the question. The *Nmax* limit of 250 was based on a previous autopsy study by Cheever and colleagues [3]. In this study, the authors recovered *S. haematobium* worms from individuals at autopsy and found the maximum number of *S. haematobium* females in infected individuals to be 250. However, we note that this was just one (rather unique) study and hence why we varied *Nmax* in our sensitivity analysis.

**Summary and General Comments**

**Reviewer’s comment 1.20:**

Overall, I found this manuscript to be well written. I find the topic interesting and it contributes substantially to the literature, especially the non-invasive nature of the methods. I have mainly minor issues, but one major concern I have is whether the two datasets included repeated measures from individual subjects. The methods mentioned before and after treatment collections of individuals. How did the authors accounted for samples who were treated, as treatment could influence density dependence.

**Authors’ response 1.20: Thank you.** We have addressed the important comment on repeated measures in **Authors’ response 1.1**. We further clarify here that in both datasets, at baseline and subsequent years, the data were collected two months prior to the administration of praziquantel, so there should be no immediate impact of treatment on density-dependent fecundity (since each child had received their last treatment either approximately six months or one year before being sampled). We have clarified in the Methods and Discussion of the revised manuscript as follows:

*“In both studies, samples were collected two months before treatment with praziquantel.”*

Reviewer #2

**Methods**

**Reviewer’s comment 2.1:** Overall the methodology is clearly described. However, the Methods should contain a brief description of how the sibship reconstruction is achieved, without having to refer to the relevant references. This is not as universally understood as immunoblotting or PCR. This would also help illuminate the subsequent discussion of the limitations of sibship reconstruction presented in the Discussion.

**Authors’ response 2.1:** We agree with the Reviewer’s suggestion, which was also raised by Reviewer # 1. We have added a brief description of the sibship reconstruction method in the Methods section of the revised manuscript (see **Authors’ response 1.10** for text)

**Results**

**Reviewer’s comment 2.2:** The Results are clearly and concisely described. No concerns.

**Authors’ response 2.2:** We thank the Reviewer for this positive appraisal.

**Conclusions**
**Reviewer’s comment 2.3:** The Conclusions are clearly and succinctly communicated, without over-interpretation. I especially appreciated the thorough and objective discussion of the study’s limitations. The potential relevance of the findings to model refinement as well as real-world efforts to control schistosomiasis are fascinating and the authors are correct to highlight these points.

**Authors’ response 2.3:** We thank the Reviewer for these appreciative and supportive comments. We are delighted that the Reviewer enjoyed the discussion of our findings.

**Editorial and Data Presentation Modifications**
**Reviewer’s comment 2.4:** Various minor typographical issues:

Line 66 – “Globally, schistosomiasis is second only to malaria in terms of socioeconomic impact [4].” Among eukaryotic pathogens, that is.
Line 76 – “if necessary”
Line 85 – “utmost importance”
Line 227 – “which is the first time”
Line 229 – “the cause of intestinal schistosomiasis”
Line 359 – “used to explore”
Line 365 – “In the event that of emerging”?
Line 367 – “rate of spread, informing”
Line 381 – “immune response, albeit”

**Authors’ response 2.4:** We thank the Reviewer for picking up on these typographical errors which we have amended in the revised manuscript.

**Summary and General Comments**

**Reviewer’s comment 2.5:** Overall, this is a well-written and highly readable manuscript which provides convincing evidence for the existence of density-dependent fecundity in Schistosoma haematobium infections. Combining molecular genotyping and traditional parasitological data with sophisticated modeling, this study is a logical extension of the authors’ previous work and should be of considerable interest to the schistosomiasis community, and to helminthologists in general.

**Authors’ response 2.5:** We are most grateful to the Reviewer for this appreciative summary and for their constructive feedback.

Reviewer #3

**Summary and General Comments**

**Reviewer’s comment 3.1:** This paper investigates an important matter in schistosome infections, namely, does the number of adult worms affect fecundity of individual females.  This is an important question, because of the intense pathogenesis of eggs laid by the females.  The work is built around some elegant genetic typing of miracidia combined with statistical analyses.
In essence, the paper presents a useful case and is worthy of consideration after some issues are addressed.

**Author’s response 3.1:** We thank the Reviewer for these appreciative words.

**Reviewer’s comment 3.2:** The proximity of the analyses to drug administration in community treatments.  The surveys were performed in sites that had been treated regularly with praziquantel.  What is the effect of PZQ on population structure in communities and in individuals?  What would be the effect of, say, killing of some, but not all, worms in humans by PZQ?

**Authors’ response 3.2:** We agree with the Reviewer that treating populations with praziquantel will likely have an effect on the population structure of schistosome communities within individuals (although we emphasise that the data were collected two months prior to treatment with praziquantel, see **Authors’ response 1.20**). This will arise for two reasons. First, because praziquantel does not kill juvenile stages of the parasite and second, because the limited reinfection periods between consecutive treatment rounds will push the demographic of the schistosome infra-populations to younger ages (i.e. more recent infections). This demographic shift could shift fecundity (that is, maximum potential fecundity or estimated egg output for a single female worm) in either direction; younger worms could be more or less fecund than older worms. The additional results presented in the Supplementary Information show that for *S. haematobium* there was very little difference in fecundity between 2012 and 2016, while for *S. mansoni* fecundity increased over the three sampling times, 2005, 2006 and 2010. However, it is important to emphasise that we controlled for the effect of sampling year in our analysis (using both additive and interactive models) and thus we are confident that our findings are robust to any potential effects of praziquantel on the schistosome infra-population structure. More generally, we discuss explicitly how the failure of praziquantel to kill all worms could interact with density-dependent fecundity and exacerbate the potential spread of drug resistance:

*“Density dependencies could enhance the spread of emerging drug resistance, due to enhanced reproduction rates in resistant or reduced-susceptibility parasites [53]. In the event of emerging resistance, understanding density-dependent processes would thus be key in understanding the likely rate of spread, informing timeframes for implementing alternative or complementary interventions, such as snail control [54] or eventually a vaccine [55, 56].”*

**Reviewer’s comment 3.3.** Is the immune or health status of the human hosts likely to confound results?  

**Authors’ response 3.3.** It is possible that immune responses may influence the number of adult worms harboured by a host and/or the fecundity of these worms. The immune mechanisms related to schistosome infection have been studied extensively in animal models and human populations. It is possible that humans develop protective immunity against schistosome infection over time, therefore influencing individual worm burden and morbidity [5], but this has been controversial [6] and the mechanisms driving this process are still not fully understood. Some studies have provided evidence that such resistance can develop slowly over time [7, 8], and one particular study found that antibody response reduced worm fecundity of *S. haematobium* [9]. Data from endemic populations also suggests that an age-associated decrease in infection may result from development of anti-parasite immunity, rather than reduced contact with water [10]. Hence, it is quite possible that the results of our phenomenological analysis are driven in part by underlying immune responses. The mechanisms driving density dependence are discussed in the revised manuscript. We also discuss how our contrasting findings—and in particular the identification of density-dependent fecundity in *S. haematobium*—with those from Cheever’s autopsy studies [3, 11] may be explained by the likely very different immune statuses of our predominantly health children compared to the cadavers used by Cheever. Relevant sections of the revised manuscript read:

*“The importance of density-dependent processes on the population biology and transmission dynamics of schistosomiasis and other helminthiases is clear and much work (including this paper) focuses on phenomenological identification, often for the purpose of integrating into mathematical models. Less clear are the biological mechanisms that drive these processes. The most straightforward explanations are putative crowding effects and competition for resources. But in schistosomiasis, more complex mechanisms may also be involved. For example, epidemiological and modelling studies have shown evidence for anti-fecundity immunity such that egg production is reduced by the host’s immune response, albeit susceptibility to new infections is sustained [58, 59].”*

*“The nature and immune status of individuals from the autopsy study is also very different from our study, since it was performed using deceased patients who had been terminally ill and had heavy worm burdens, whereas our study was conducted primarily using data from children attending primary schools.”*

**Reviewer’s comment 3.4.** Recently, there have been some imaging and immunological methods suggested to estimate worm burdens in the host.  perhaps some reference to these could be made, if only to comment on their usefulness.

**Authors’ response 3.4.** We agree with the Reviewer that the use of immunological techniques, particularly CAA and CCA has been used investigate the relationship between circulating *S. mansoni* antigens and egg counts. In 2001, Polman et al. [12] found that the relationship between these two was not proportional (indicative of density-dependent fecundity), although it remains unclear how measured antigen levels relate to worm burdens, which complicates interpretation. Radiologic imaging, especially through ultrasound examination, has also been fundamental for morbidity assessment of organ damage but we are not aware of it being used to estimate worm burdens. We refer to the findings from Polman et al. [12] in the revised manuscript as follows:

*“In 2001, taking a different approach, Polman et al. [47] found that the relationship between circulating S. mansoni antigens and egg counts was not proportional, potentially indicative of density-dependent fecundity. However, the interpretation of this relationship relies on the assumption that measured antigen levels relate to worm burdens in a linear or proportional manner, which remains unclear.”*

**References**

1.	Norton AJ, Gower CM, Lamberton PHL, Webster BL, Lwambo NJS, Blair L, et al. Genetic consequences of mass human chemotherapy for Schistosoma mansoni: Population structure pre-and post-praziquantel treatment in Tanzania. American Journal of Tropical Medicine and Hygiene. 2010.

2.	Gower CM, Gouvras AN, Lamberton PH, Deol A, Shrivastava J, Mutombo PN, et al. Population genetic structure of Schistosoma mansoni and Schistosoma haematobium from across six sub-Saharan African countries: implications for epidemiology, evolution and control. Acta tropica. 2013;128(2):261-74.

3.	Cheever AW, Kamel IA, Elwi AM, Mosimann JE, Danner R. Schistosoma mansoni and S. Haematobium infections in Egypt ii. Quantitative parasitological findings at necropsy. The American Journal of Tropical Medicine and Hygiene. 1977;26:702-16.

4.	GBD 2015 Disease and Injury Incidence and Prevalence Collaborators. Global, regional, and national incidence, prevalence, and years lived with disability for 310 diseases and injuries, 1990-2015: a systematic analysis for the Global Burden of Disease Study 2015. Lancet. 2016;388(10053):1545-602.

5.	Mawa PA, Kincaid-Smith J, Tukahebwa EM, Webster JP, Wilson S. Schistosomiasis morbidity hotspots: roles of the human host, the parasite and their interface in the development of severe morbidity. Frontiers in Immunology. 2021;12:751.

6.	Warren KS. Regulation of the prevalence and intensity of schistosomiasis in man: immunology or ecology? The Journal of infectious diseases. 1973:595-609.

7.	Fitzsimmons CM, Jones FM, de Moira AP, Protasio AV, Khalife J, Dickinson HA, et al. Progressive cross-reactivity in IgE responses: an explanation for the slow development of human immunity to schistosomiasis? Infection and immunity. 2012;80(12):4264-70.

8.	Karanja DM, Hightower AW, Colley DG, Mwinzi PN, Galil K, Andove J, et al. Resistance to reinfection with Schistosoma mansoni in occupationally exposed adults and effect of HIV-1 co-infection on susceptibility to schistosomiasis: a longitudinal study. The Lancet. 2002;360(9333):592-6.

9.	Mitchell KM, Mutapi F, Savill NJ, Woolhouse ME. Protective immunity to Schistosoma haematobium infection is primarily an anti-fecundity response stimulated by the death of adult worms. Proc Natl Acad Sci U S A. 2012;109(33):13347-52.

10.	Mitchell KM, Mutapi F, Savill NJ, Woolhouse MEJ. Explaining observed infection and antibody age-profiles in populations with urogenital schistosomiasis. PLoS Computational Biology. 2011;7:e1002237.

11.	Cheever AW. Density-dependent fecundity in Schistosoma mansoni infections in man: a reply. Transactions of the Royal Society of Tropical Medicine and Hygiene. 1986;80:991-2.

12.	Polman K, De Vlas SJ, Van Lieshout L, Deelder AM, Gryseels B. Evaluation of density-dependent fecundity in human Schistosoma mansoni infections by relating egg counts to circulating antigens through Deming regression. Parasitology. 2001;122:161-7.
